# A TRilogy of ATR’s Non-Canonical Roles Throughout the Cell Cycle and Its Relation to Cancer

**DOI:** 10.3390/cancers16203536

**Published:** 2024-10-19

**Authors:** Yoon Ki Joo, Carlos Ramirez, Lilian Kabeche

**Affiliations:** 1 Department of Molecular Biophysics and Biochemistry, Yale University, New Haven, CT 06511, USA; 2 Yale Cancer Biology Institute, Yale University, West Haven, CT 06516, USA

**Keywords:** ATR, DNA damage response (DDR), cancer therapy, clinical trials, non-canonical roles, nuclear membrane, mechanical forces, mitosis, chromosome segregation, PML bodies

## Abstract

Ataxia Telangiectasia and Rad3-related protein (ATR) is an apical kinase of the DNA Damage Response (DDR) pathway responsible for detecting and resolving damaged DNA. Because cancer cells depend heavily on the DNA damage checkpoint for their unchecked proliferation and propagation, ATR has gained enormous popularity as a cancer therapy target in recent decades. Yet, ATR inhibitors have not been the silver bullets as anticipated, with clinical trials demonstrating toxicity and mixed efficacy. To investigate whether the toxicity and mixed efficacy of ATR inhibitors arise from their off-target effects related to ATR’s multiple roles within and outside the DDR pathway, we have analyzed recently published studies on ATR’s non-canonical roles. Recent studies have elucidated that ATR plays a wide role throughout the cell cycle that is separate from its function in the DDR. This includes maintaining nuclear membrane integrity, detecting mechanical forces, and promoting faithful chromosome segregation during mitosis. In this review, we summarize the canonical, DDR-related roles of ATR and also focus on the non-canonical, multifaceted roles of ATR throughout the cell cycle and their clinical relevance. Through this summary, we also address the need for re-assessing clinical strategies targeting ATR as a cancer therapy based on these newly discovered roles for ATR.

## Simple Summary

In this review, we introduce and summarize the canonical roles of Ataxia Telangiectasia and Rad3-related protein (ATR) in the DNA damage response (DDR) pathway. More importantly, we also summarize the most recent discoveries of ATR’s non-canonical roles in regulating nuclear membrane integrity, mechanical force, chromosome segregation, and its role in PML bodies and discuss how they are relevant to the current progress of ATR inhibitors in clinical trials.

## 1. Introduction

Despite decades of research, cancer remains one of the most prevalent and difficult diseases to treat, with 600,000 people in the U.S. estimated to suffer cancer-associated deaths in 2024 alone [1]. While there are many reasons that make cancer difficult to treat, one reason is its heterogeneity, where cancer cells can be different from each other even within the same tumor. One source of variation between cancer cells is in their karyotypes. Consequently, when it was discovered that over 90% of solid tumors possess aneuploidy phenotype, a state where cells harbor aberrant copies of chromosomes, targeting aneuploidy and chromosomal instability (CIN) became an extremely popular subject of research in cancer biology [2,3,4]. 

Aneuploidy can lead to complex genome rearrangements resulting from CIN, often increasing the DNA damage burden and challenges in replication [5,6,7,8]. Therefore, many cancer cells have increased reliance on the DDR pathway because of (a) increased DNA damage/replication stress and (b) faulty checkpoints to support proliferation in the presence of DNA damage [9,10]. Therefore, one cancer therapy strategy that has gained popularity is taking advantage of the synthetic lethality of DDR components. This strategy inhibits DDR components in cancer cells that already have impaired DNA damage repair, pushing cancer cells past the point of viability and selectively killing cancer cells [11,12]. As such, ever since Ataxia Telangiectasia Mutated and Rad3-related protein (ATR) was discovered to be a crucial component in the DDR pathway, developing strategies to target ATR have gained significant popularity as a cancer therapy [13,14,15,16]. A host of different ATR inhibitors have been developed over the past decade to try to attack genomically unstable tumors that lack full DDR activity [11,16,17,18,19,20]. However, while some of these inhibitors have been tested in phase-1 and -2 clinical trials, they are still not used as standard treatments because of high toxicity and off-target effects [17,18,19,20]. 

Mutations in ATR can give rise to Seckel syndrome, a rare genetic condition characterized by dwarfism, microcephaly, radial dislocation, dysplasia, and intellectual disability with decreased life expectancy (report from the National Organization for Rare Disorders, NORD). Seckel syndrome affects about 1 in 10,000 individuals and is considered a rare disease by the NIH and treatment is limited to supportive therapy (report from the NORD). Moreover, mutations in ATR are also associated with additional conditions including: ataxia, telangiectasia, mental disabilities, and overall nervous system defects, all of which occur in tissues that are not considered highly proliferative, which seems paradoxical to the established role of ATR as a cell cycle checkpoint protein [13,21,22,23,24,25,26,27,28]. Although ATR has been extensively studied as part of the DNA damage response pathway, its disease etiology remains unclear, especially as increased DNA damage alone does not fully recapitulate the phenotype of ATR loss [29]. The failure of ATR inhibitors in clinical trials, coupled with the still unknown links between the pathophysiology of ATR in patients with Seckel syndrome and other defects, has suggested that there are other incompletely understood roles of ATR that have been previously overlooked. In this review, we will briefly summarize our current understanding of the canonical roles of ATR, recent discoveries describing non-canonical ATR activity, and the multifaceted role of ATR as a master regulatory protein. 

## 2. Canonical Roles of ATR 

Ever since the importance of ATR in maintaining genome integrity through the DDR pathway was elucidated, a significant amount of research has been done to uncover the mode of recruitment, activation mechanism, and downstream pathways of ATR. Due to its importance and popularity, the canonical roles of ATR have been reviewed extensively elsewhere [30,31,32]. Therefore, this section serves to summarize and highlight relevant information on ATR’s canonical roles to draw meaningful comparisons in a later section, where we discuss more recently identified non-canonical roles of ATR. In this section, we give a brief overview of how ATR is recruited, what regulates its activation, and what downstream pathways it triggers in the context of DDR. 

### 2.1. Canonical Recruitment and Activation of ATR

#### 2.1.1. Initial Recruitment of ATR 

ATR is a member of the phosphatidylinositol 3-kinase-related kinase (PIKK) protein family, which is highly conserved across eukaryotes. The PIKK kinase family includes important proteins for cell cycle regulation and DNA repair signaling, including ATM and DNA-PK, which often redundantly share downstream substrates with ATR [33,34]. Despite the structural and biochemical similarities between ATR, ATM, and DNA-PK, their modes of recruitment and activation are vastly different [35,36,37]. While ATM and DNA-PK are predominantly recruited to DNA double-stranded breaks (DSBs), ATR is recruited to DNA single-stranded breaks (SSBs) and single-stranded DNA (ssDNA) lesions [38]. However, whether ATR activation is a consequence of initial ssDNA lesions or the product of indirect activation by other components of the DDR pathway has been a topic of active research [39,40]. 

ssDNA lesions can occur naturally, even in cells with no exposure to DNA damaging agents, either as a consequence of fork stalling due to oncogenic activation or due to complex replication DNA sequences [32]. ssDNA structures can also result from DNA damaging agents, resection of DSBs, or nucleotide excision repair intermediates [41,42,43]. Importantly, these ssDNA serve as a scaffold for cofactors that recruit and activate ATR. ssDNA structures are rapidly coated by replication protein-A (RPA), which is necessary for initial ATR interaction protein (ATRIP) recruitment, an important subunit of ATR activation [36,44]. Once ssDNA is coated with RPA, ATRIP binds RPA to allow for proper docking and subsequent activation of ATR, which is mediated through ATRIP’s ATR activating domain (AAD) [40]. While ATRIP alone is sufficient to induce basal kinase activity of ATR, additional activators are necessary to significantly increase its kinase activity for efficient signaling and completion of its regulatory feedback loop [30,45].

#### 2.1.2. Activation of ATR Through TopBP1 and ETAA1 

**Figure 1:**
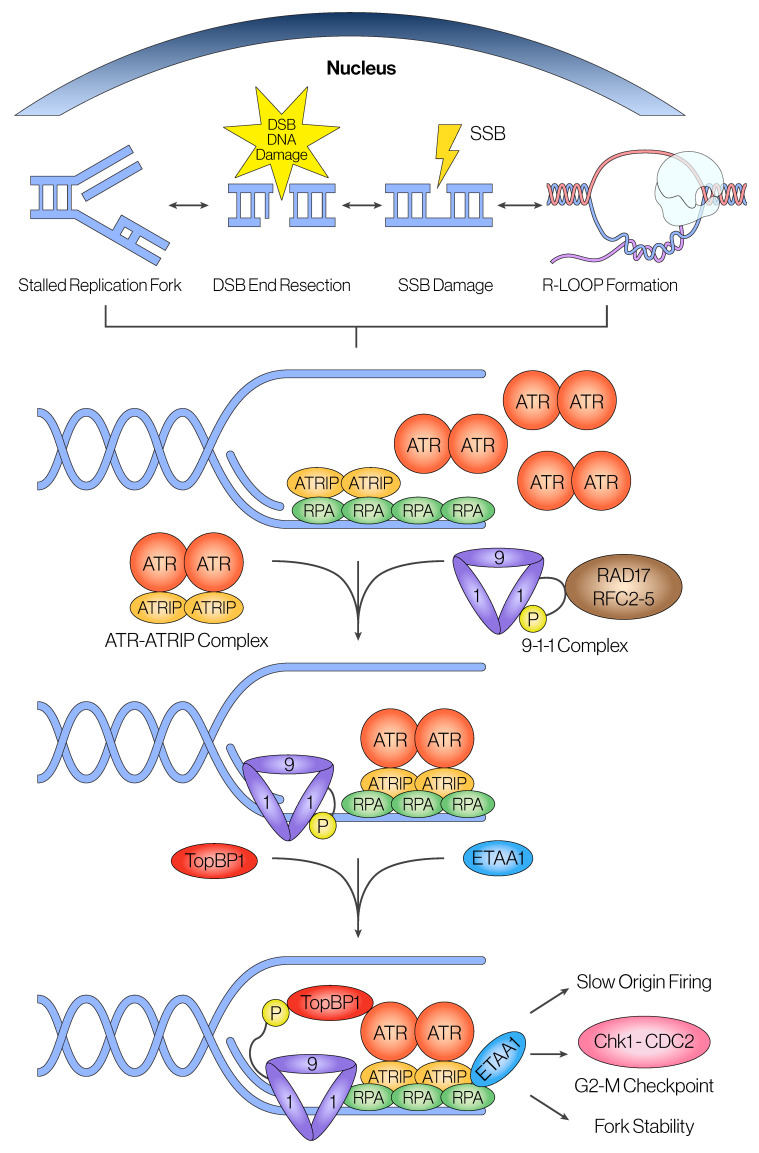
**Mechanism of ATR recruitment and activation in interphase.** ATR can be recruited and activated by different forms of DNA damage such as single-stranded breaks (SSBs), double-stranded break (DSB) end resection, or stalled replication forks during replication stress. Regardless of the type of damage, a shared DNA structure required for ATR recruitment is the formation of ssDNA gaps with a 5′ end. This common structure is required for RPA accumulation on ssDNA and recruitment of ATR binding partner ATRIP to form the ATRIP-ATR complex. Independently, the 9-1-1 complex clamp gets loaded onto the site by the RFC-Rad17 complex. Additionally, the ATR activator TopBP1 is brought into proximity by interaction of its BRCT domains and the phosphorylated C-terminal tail on the 9-1-1 complex clamp. While TopBP1 alone is able to yield full ATR activation by using its ATR activating domain (AAD), the novel protein ETAA1 has also been shown to activate ATR during replication stress. Lastly, full ATR activation leads to phosphorylation of the downstream kinase Chk1 to promote the strong G2/M cell cycle arrest checkpoint. Downstream activation of other ATR effectors can promote fork stability locally at sites of replication stress and globally on slow origin firing before mitotic entry.

So far, two ATR activators have been identified: DNA Topoisomerase II -Binding Protein 1 (TopBP1) and Ewing Tumor-Associated Antigen 1 (ETAA1). TopBP1 requires additional cofactors for its proper recruitment and activation in response to ssDNA stimuli. The first co-factors to be discovered were the RAD17-replication factor C (RFC) complex and the RAD9-RAD1-HUS1 (9-1-1) complex that acts as a ring-like clamp on ssDNA [46,47]. Simplistically, the RFC complex first recognizes RPA-coated ssDNA and loads the ring-like 9-1-1 complex onto the DNA strand (Figure 1) [48]. Once the 9-1-1 complex is loaded, phosphorylation of RAD9’s C-terminal tail allows for phosphopeptide recognition by TopBP1’s BRCT domains I and II, which positions its ATR activating domain (AAD) to interact with and activate ATR [49,50,51].

While it has been well established that TopBP1-ATR interaction is crucial for full checkpoint activation in response to both DNA damage and replication stress [52,53], new evidence provided further insight into the sequence of events and mechanism that regulate this interaction when there is a different ssDNA structure. For instance, the MRN complex has recently been shown to be an important mediator of TopBP1 recruitment when the initial stimuli are from dsDNA-ssDNA junctions. Under this circumstance, researchers show that the MRN becomes the recruiter for TopBP1 at the junction site regardless of the 9-1-1 clamp, which relegates the recruiter role of the 9-1-1 complex to be only an activator of TopBP1 [54]. Furthermore, work from Yoo and colleagues [55] expanded the mechanisms behind DSB initial signaling and the regulation of TopBP1. Here, they discovered that CtIP works as a third co-factor necessary in the interaction between NBS1, a member of the 9-1-1 complex, and TopBP1. Since CtIP is partially recruited by the MRN complex after DSB recognition, the regulatory role of CtIP on NBS1 serves as a bridge between the dsDNA damage pathway and the ATR checkpoint activation by yielding full TopBP1-ATR signaling after dsDNA damage recognition. Interestingly, researchers have shown that BACH1/FANCJ helicase has a novel direct interaction with TopBP1 that is critical to ensure RPA loading on ssDNA structures on stalled replication forks [56]. This distinct mechanism is unique to S-phase after replication stress and poses an unexpected model for TopBP1 regulation and activation by BACH1 to ensure checkpoint activation during replication stress. Overall, further research is needed to understand the differences in stimuli that trigger each unique form of TopBP1 regulatory activation and recruitment to DNA in a case-dependent manner. 

More recently, the Mailand group and the Cortez group have co-discovered that ETAA1, which is only found in mammals, is a TopBP1-independent ATR activator [57,58,59]. ETAA1 has a unique ability to directly bind to RPA molecules coating ssDNA regions and colocalize with ATR-ATRIP complexes during replication stress. This poses a distinctive mechanism of recruitment for ETAA1 compared to that of TopBP1, which requires the 9-1-1 complex [58]. Like TopBP1, ETAA1 also possesses an ATR activating domain (AAD) located at the N-terminus, which mediates the ATR-ETAA1 interaction [59]. Through its AAD, ETAA1 interacts with ATR independently of TopBP1, promoting its activation at the S/G2 replication checkpoint [59]. Recently, researchers have shown a crucial role for ETAA1 in preventing mitotic defects uniquely associated with under-replicated DNA and that replication-dependent phosphorylation events on its AAD strictly regulate this role [60]. This regulation of ETAA1’s AAD occurs during normal S-phase and is highly stimulated following induction of DNA replication stress, suggesting that there is a temporal division of labor between ETAA1 and TopBP1: while TopBP1 is a dominant ATR activator during ATR-mediated repair of ssDNA breaks, ETAA1 becomes the main activator of ATR during S-phase to promote chromosomal stability [59,60]. Overall, these two different ATR activators might be responsible for promoting ATR activity in a task-dependent manner.

### 2.2. Canonical ATR Downstream Pathways 

#### 2.2.1. ATR-Chk1 Axis and the G2/M Checkpoint 

**Figure 2:**
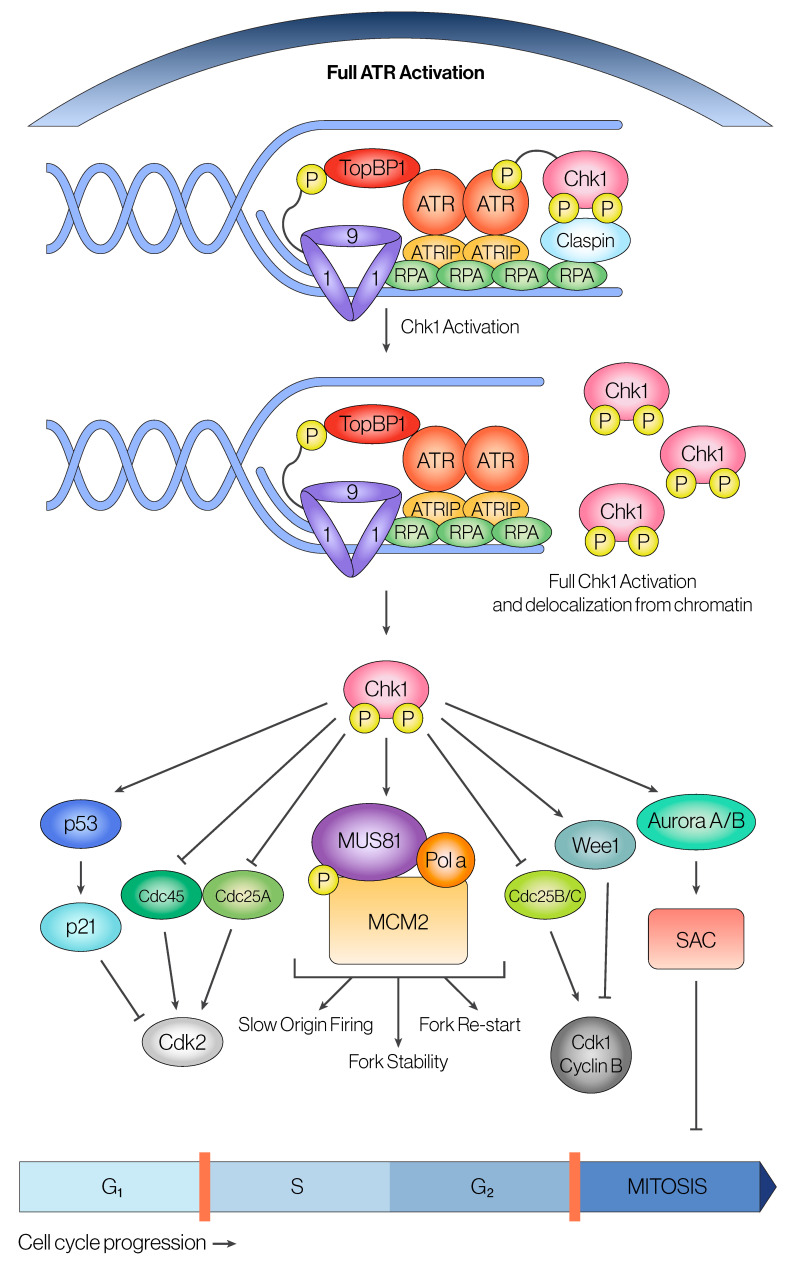
**The ATR-Chk1 signaling axis is a master regulator of cell cycle progression and DNA replication.** Complete ATR activation leads to phosphorylation of different downstream effectors located at the replication fork; however, the major ATR effector kinase is Chk1. Before activation, Chk1 is bound to chromatin by its adaptor protein, Claspin, which enhances the ATR-Chk1 interaction. ATR phosphorylation leads to delocalization of Chk1 from chromatin, yielding full kinase activity and the downstream signaling cascade of numerous Chk1 substrates. Primarily, Chk1 regulates cell cycle transitions by inhibiting the activation of the CDKs, which is achieved through inhibition of the CDC25 phosphatase family as well as activation of the Wee1 kinase family. The ATR-Chk1 signaling axis is the fundamental regulator of the cell cycle, and most evidence agrees that Chk1 activation is necessary to ensure fork stability, fork re-start at stalled replication forks, and slowing origin firing during replication stress. Yet, many of the precise mechanisms and effectors behind this regulation remain poorly understood.

Once ATR signaling becomes fully activated, several downstream reactions and substrate interactions take place. One of the most well-studied signaling roles of ATR activity is to temporarily halt the cell cycle progression to prevent premature entry into mitosis with unrepaired or unreplicated DNA [61]. Following activation, ATR phosphorylates its major downstream effector, Chk1 kinase, via the Claspin mediator protein to increase its kinase activity and delocalize Chk1 from chromatin to generate a global response [62] (Figure 2). Claspin has to be recruited by the 9-1-1 complex, which requires phosphorylation by the ATR-TopBP1 axis to create signal amplification and feedback-loop specificity to regulate checkpoint arrest [63]. Activation of Chk1 results in a series of phosphorylation events that inhibit CDC25 phosphatase and promote Wee1 kinase activation, which are two direct upstream regulators of Cyclin-Dependent Kinase 1 (CDK1) that remove or phosphorylate the major inhibitory phosphorylation site on CDK1, Tyr15 [64,65,66,67,68]. Since CDK1 activity is essential for mitotic entry, Chk1’s kinase activity ultimately results in cell cycle arrest through regulation of CDK1 [69,70,71]. Prolonged cell cycle arrest through the ATR-Chk1 axis could also result in apoptosis in a p53-dependent manner [72]. Apart from its role at the G2/M checkpoint, the ATR-Chk1 axis has also been shown to be present during S-phase and is important for regulating the S/G2 transition [73,74]. 

#### 2.2.2. ATR in Regulation of Origin Firing and Fork Progression During S-Phase

While regulation of CDK1 activity at the G2/M and S/G2 checkpoint through the ATR-Chk1 axis is one of the most prominent roles of ATR, ATR is also important during the S-phase to reduce origin firing and slow down fork progression during DNA replication. The exact mechanism of how ATR is able to reduce origin firing and fork progression is not well understood [75,76]. The absence of ATR activity is deleterious for the re-stabilization of replication forks under DNA damage conditions [75,76]. Even without the presence of exogenous DNA damaging agents, ATR is important for regulating origin firing density and initiation during normal cell cycling [77,78,79]. Work from the Blow lab suggests that this regulation occurs via Chk1 and CDK2-Cyclin E [78]. Moreover, recent work also shows that many ATR substrates are located at the replication fork, including Pol ε, PCNA, RPA1, RPA2, the minichromosome maintenance complex, and several other polymerases [75,80,81,82,83]. Thus, it is possible that ATR regulates origin firing via multiple diverse mechanisms. Perturbations to ATR that decrease its activity can cause the delocalization of these factors from the replication fork, which may lead to unresolved fork stalling and under-replication phenotypes that have been associated with ATR inhibition at the fork [75]. Likewise, ATR can inhibit origin firing indirectly through post-translational modifications on chromatin, such as by phosphorylating lysine methyl transferase MLL, which directly methylates H3K4 nucleosomes [84]. Since H3K4 methylated nucleosomes cannot interact with CDC45 origin helicase, this blocks origin initiation on chromatin areas affected by DNA damage [85]. 

The role of ATR in replication integrity is more intricate than a simple regulatory inhibition and rehabilitation fork kinase. Particularly, there has been growing evidence of ATR promoting, instead of inhibiting, late replication origin firing of dormant origin sites when there is exogenous replication stress [86]. The currently proposed mechanism for this paradoxical role of ATR is the discovery of ATR-mediated phosphorylation on MCM2 at the replication fork, which was previously uncharacterized [83,87,88,89,90,91]. Researchers showed that this phosphorylation at this site of MCM2 allows Polo-like Kinase 1 (PLK1) to be recruited to the fork by binding its polo box domain [78,91]. The presence of PLK1 acts as a repressor of the ATR-Chk1 checkpoint axis active during the S-phase when the cell faces replication stalling after drug treatment [86]. Yet, this proposes a contradictory role of ATR function that seems highly inefficient for a cell suffering from replication stress conditions. Although paradoxical, this could explain the duality of ATR in this system—by inhibiting global origin firing through the activation of the Chk1-CDC25 checkpoint, ATR ensures that replication under stress is slowed before a cell commits to mitosis [92]. At the same time, ATR recruitment of PLK1 to the fork promotes local dormant origins to initiate replication by inhibiting local Chk1 S-phase checkpoint blockade in an attempt to complete DNA replication on time while troubled forks recover [86,93,94]. 

The field of ATR-mediated genome maintenance has gained significant advances in understanding the potential roles of ATR in signaling and cross-talking with different pathways to protect genome integrity. More information is needed to fully elucidate ATR activation and downstream functions, especially as the number of known ATR-interacting proteins grows. Many lingering questions still do not fit the canonical model for ATR activity, alluding to the presence of non-canonical forms of ATR activity that are required for other cellular physiological processes that could explain the complexity seen in ATR-deficient cell phenotypes. 

## 3. Non-Canonical Roles of ATR 

In the previous section, we described how ATR is recruited and activated, as well as its downstream pathways following DNA damage. ATR requires critical factors for its recruitment and activation—namely, RPA, ATRIP, and TopBP1/ETAA1. Because these factors are mostly present at sites of DNA damage, it may seem counterintuitive to assume that ATR could be recruited to sites without DNA damage, let alone remain active at those sites. 

However, more recent evidence in the past decade shows that ATR is not limited to the DDR pathway. In fact, ATR seems to have multiple roles that are indirectly or even completely unrelated to its roles in the DDR pathway. In this section, we focus on the non-canonical, multifaceted roles of ATR, specifically in mitosis and at the nuclear membrane. 

### 3.1. ATR in Mitosis 

When DNA damage occurs during mitosis, γH2AX can be robustly detected, suggesting that the machinery that detects and marks sites of DNA damage such as ATR, ATM, and DNA-PK, remains active [95,96]. Repair of such damage, however, is largely inactivated and mostly occurs after cells exit mitosis and enter G1 [97,98,99]. These findings imply that the DDR pathway is somehow rewired during cell division and the classical components of the DDR pathway might have a mitosis-specific role that is distinct from their roles during interphase. Recent evidence from many labs suggests that ATR is not only active during mitosis but that its downstream pathway is also rewired. There seem to be three main differences between the ATR pathway in the DDR and mitosis: its recruitment site, activation mechanism, and downstream effects. 

#### 3.1.1. Recruitment of ATR During Mitosis

**Figure 3:**
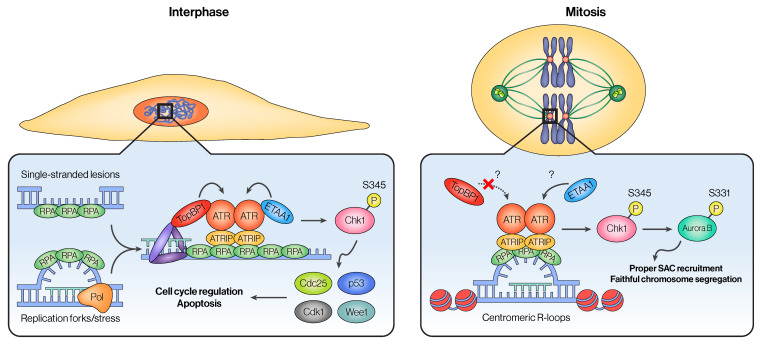
**Comparison of ATR activation and signaling pathway in interphase and in mitosis.** As described extensively in the previous section, ATR is primarily activated through the creation of single-stranded DNA breaks (SSBs) or stalled replication forks during interphase, which act as recruitment sites for ATRIP, an essential ATR binding partner. Once ATR is recruited to these sites, additional factors, including the 9-1-1 complex, TopBP1, and ETAA1, are able to fully activate ATR and the downstream signaling cascade, mainly through Chk1. The primary role of active Chk1 in this context is to arrest the cell cycle until the damaged DNA is repaired or to promote apoptosis if the degree of DNA damage is beyond repair. In contrast, ATR in mitosis is primarily recruited to the centromeres through centromeric R-loops, which are likely formed by transcription of the centromeric regions. Once activated, ATR activates Chk1 as it does during interphase. However, active Chk1 in mitosis primarily phosphorylates and activates Aurora kinase B and also promotes recruitment of essential spindle assembly checkpoint (SAC) components to the kinetochore to promote faithful chromosome segregation.

While ATR is normally recruited to stalled replication forks or DNA single-stranded breaks (SSBs) in the context of DNA damage, ATR is also recruited to centromeric R-loops during mitosis (Figure 3) [100,101,102]. R-loops are three-stranded DNA-RNA hybrid structures that displace an unpaired, single-stranded DNA, which is formed either in cis through transcription or in trans by hybridization of mRNA transcribed elsewhere [103,104,105]. While transcription is mostly silenced during mitosis, mitotic cells still maintain global albeit low levels of transcription, notably at the centromeres [106,107]. Because the single-stranded DNA displaced from R-loops is coated with RPA, these centromeric R-loops serve as a recruitment site for ATR during mitosis, where ATR appears as distinct foci that colocalize with centromeric markers [102]. One alternative explanation as to why ATR is seen at the centromeres is that centromeres are known to be fragile and susceptible to breaks, potentially suggesting that ATR is simply being recruited to sites of DNA damage. However, factors that are commonly recruited to such breaks, including ATM, have not been shown to form such distinct foci, suggesting that ATR’s localization to centromeres is distinct from its role in repairing DNA damage [108,109]. Other studies have also shown that DDR proteins localize to telomeric regions during mitosis to promote mitotic DNA synthesis (MiDAS) following break-induced replication stress, but it is unclear if ATR localizes to these sites during mitosis or affects centromeric transcription [110]. Despite the lingering questions, these studies all imply that SSBs and stalled replication forks are sufficient but not necessary for ATR recruitment, providing evidence that ATR could function outside the context of DDR.

#### 3.1.2. Activation of ATR in Mitosis

Once recruited, ATR has to be activated to perform its function fully. Two activators of ATR have been identified so far: TopBP1 and ETAA1 [53,57]. While its major function in DNA damage repair happens during interphase, TopBP1 has been implicated in promoting mitotic fidelity as well. In mitosis, TopBP1 localizes to the centrosomes and sites of DSBs, where it mediates a host of processes, including chromosome segregation, mitotic progression, and MiDAS [111,112,113]. Some studies also show that TopBP1 also localizes to the chromosome core and co-localizes with ATR, although these studies were performed in the context of meiosis [114,115]. While these studies show robust evidence that TopBP1 plays a vital role in mitosis, whether ATR is part of the pathway that links TopBP1 to mitotic fidelity is less clear and requires further investigation. Compared to TopBP1, there is much less literature regarding the localization and function of ETAA1 in mitosis. This might partly be due to the fact that ETAA1 was discovered more recently and that its role in the DDR is much less prominent than TopBP1 [59,116]. Interestingly, however, ETAA1, not TopBP1, was suggested to be the major activator of mitotic ATR. One phosphoproteomics study specifically focusing on mitotic cells showed that disturbing the interaction between ETAA1 and ATR, but not TopBP1 and ATR, caused changes in pathways that are specific to mitosis, including spindle assembly checkpoint (SAC) and chromosome segregation [117,118]. While this study gives valuable insight into how ETAA1 and TopBP1 contribute to ATR activation in a mitosis-specific manner, their method to abolish ETAA1-ATR interaction is through deletion of the ATR activating domain (AAD) [119] through CRISPR-induced genome editing, which may introduce confounding effects from interphase. Nonetheless, these studies provided important insights into the differential roles of TopBP1 and ETAA1 in activating ATR at different stages of the cell cycle. 

#### 3.1.3. Downstream Effects of Mitotic ATR Activity

While it is arguably the most important question to answer, investigating the downstream effects of ATR, specifically in mitosis, is quite challenging. To properly answer the question, ATR has to be acutely inhibited or depleted after the cells enter mitosis, and the effects have to be measured before the mitotic exit. While it is possible to genetically knockdown or partially knockout ATR, these strategies will introduce confounding effects from interphase [120,121]. Tagging ATR with inducible degrons is also not a viable option since ATR requires its N-terminus for interaction with ATRIP and subsequent recruitment to RPA [122] (although binding of ATR to ATRIP is dispensable for its kinase activity [40]) and the C-terminus for its kinase activity [123]. Therefore, most studies on the mitotic role of ATR rely on small molecule inhibitors or manipulation of its upstream activator (TopBP1 or ETAA1) and its direct downstream effector kinase, Chk1 [102,117,124]. Kabeche et al. and Bass and Cortez used two different approaches in their studies, where Kabeche et al. used small-molecule inhibitors and RNase H1 to acutely inhibit ATR activity in mitosis, while Bass and Cortez utilized a system expressing ETAA1 lacking the ATR activating domain (ETAA1-ΔAAD). Intriguingly, findings from both approaches agree that disrupting ATR activity in mitosis causes a decrease in Aurora kinase B (AurKB) activity, a major player in mediating the kinetochore–microtubule attachments [102,117]. Zachos et al. also showed that Chk1 is crucial to Aurora B and SAC activity and additionally showed that Chk1 can phosphorylate Aurora B at Ser331 in vitro [124]. While the possibility that Chk1 directly phosphorylates Aurora B in mitosis is very plausible from the in vitro study, further validation is required to determine whether Aurora B is a direct Chk1 substrate in vivo. The study further showed that Chk1 localizes to the kinetochores during mitosis, providing evidence for activation of Chk1 through centromere-localized ATR. Based on these findings, the prevailing theory seems to be that ATR still activates Chk1 during mitosis, which in turn enhances Aurora B activity to promote proper chromosome segregation. 

### 3.2. Other Non-Canonical Roles of ATR 

#### 3.2.1. ATR and the Nuclear Envelope

**Figure 4:**
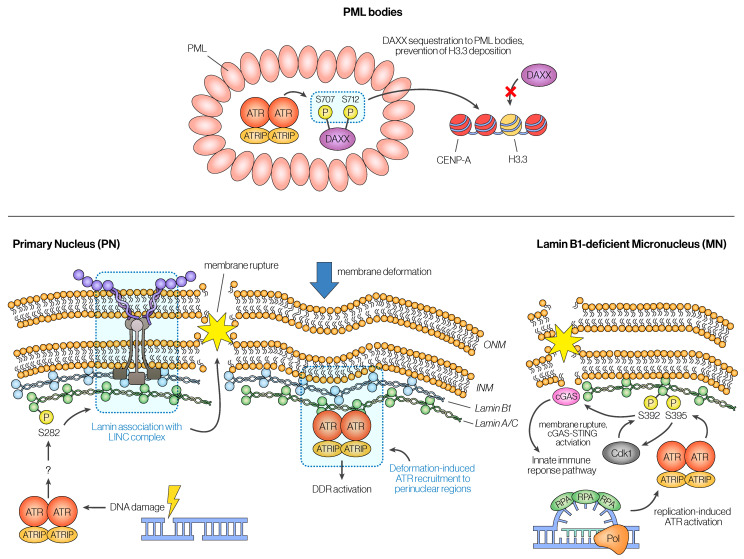
**ATR’s role at the nuclear membrane and at PML bodies.** Other than its canonical roles described in Figure 1 and Figure 2, ATR also has more recently discovered non-canonical roles in interphase. ATR can associate with phase-separated structures known as the Promyelocytic Leukemia (PML) bodies, where it can phosphorylate another PML-associated protein, DAXX, and sequesters it to the PML bodies. Since DAXX is a histone chaperone responsible for depositing H3.3 histones, sequestration of DAXX at PML bodies prevents H3.3 deposition and causes changes in the distribution of CENP-A and H3.3 nucleosomes at the centromeres. ATR also has intriguing roles in the nuclear envelope, where it can promote the rupture of both the primary nucleus (PN) and the micronucleus (MN). Mechanistically, ATR can promote phosphorylation of Lamin A/C either directly or indirectly. In the PN, phosphorylation of Lamin A/C through ATR promotes Lamin A/C association with the LINC complex, which leads to rupture; in the MN, phosphorylation of Lamin A/C by ATR primes CDK1 to further phosphorylate Lamin A/C at another site, which promotes disassembly of the micronuclear envelope.

Similarly to the surprising finding that ATR is active in mitosis, recent work has identified that ATR is active at the nuclear membrane, where studies have recently identified emerging roles for ATR in nuclear disassembly and safeguarding nuclear integrity against mechanical stress (Figure 4). 

Nuclear envelope rupture can be caused spontaneously or through external stress [125,126]. Surprisingly, ATR seems to play an important role in both cases. While the relationship between ATR and the nuclear envelope might be puzzling, ATR’s localization to the nuclear envelope has been shown previously. Two separate studies have reported that a fraction of total ATR is enriched at membranes [127], and more could be recruited to the nuclear membrane upon mechanical stress [128,129]. While these studies did not focus on the phosphorylation of the nuclear lamina itself, two subsequent studies showed that ATR at the nuclear membrane is responsible for phosphorylation of the nuclear Lamin A/C, which is crucial for Lamin integrity and nuclear rupture [130,131]. Kovacs et al. showed that ATR-dependent phosphorylation of Lamin A Ser282 plays an important role in DNA-damage-induced nuclear envelope rupture by mediating the anchoring between the cytoskeleton and the nuclear envelope [130]. Joo et al. also showed that ATR also promotes nuclear envelope rupture, although they identified a separate Lamin A site, Ser395, that mediates this effect [131]. In contrast to Kovacs et al., Joo et al. showed that ATR’s kinase activity promotes the rupture of micronuclei that are formed due to a chromosome missegregation event [132]. In this study, Joo et al. suggested that ATR directly phosphorylates Lamin A at Ser395, and this phosphorylation, in turn, allows CDK1 to efficiently phosphorylate Ser392, leading to micronuclei rupture [131,133,134,135]. While Kovacs et al. did not claim that Lamin A/C is a direct ATR substrate, the possibility remains and requires further investigation. The two studies, Kovacs et al. and Joo et al., raise the interesting question of whether the proposed mechanisms for each study are mutually exclusive. Does ATR also phosphorylate Lamin A/C at the primary nucleus (PN) at Ser395 as it does in the micronucleus (MN)? Does ATR also associate with mechanosensory machinery to phosphorylate the Lamin A/C at Ser282? While properly answering these questions requires further investigation, these mechanisms might indeed be distinct from one another due to the differences between the PN and MN—for instance, MN commonly has deficiencies rarely seen in PN, such as the lack of nuclear pore complexes (NPCs) and Lamin B1 deficiency [136,137,138]. Regardless, both studies, as well as previous studies, demonstrate that ATR has a non-canonical association with the nuclear membrane unrelated to its conventional role in the DDR pathway. 

#### 3.2.2. ATR and PML Bodies

PML nuclear bodies are diverse structures that house a host of different proteins and are thought to play an important role in chromatin regulation, transcription, and DNA damage response [139,140,141]. One of the main ways that PML bodies recruit myriads of partner proteins is through a type of post-translational modification called SUMO, which can be recognized and bound by SUMO Interacting Motifs (SIM) that are present in these partner proteins [142,143]. Interestingly, ATR regulation is also dependent on SUMOylation, where the ATR/ATRIP complex is recruited to the sites of DNA damage partly through SUMOylation of ATRIP [144]. Therefore, not surprisingly, ATR, along with other DDR components, has also been found to be present and active in PML bodies [145,146], likely through its interaction with SUMO [146,147]. Recent work has shown that ATR can directly phosphorylate DAXX, a histone H3.3 chaperone that also localizes to PML bodies on its C-terminal domain. This, in turn, leads to a relocalization of DAXX from PML bodies to centromeres and, in turn, modulates CENP-A and histone H3.3 occupancy at centromeres [16,146,148]. This finding provides new evidence for how ATR’s DDR-unrelated role can have effects on genome stability during mitosis since the regulation of CENP-A occupancy at centromeres plays an important role in faithful chromosome segregation [149].

## 4. ATR and Clinical Trials

Several ATR inhibitors alone or in combination are currently being tested in the clinic. Overall, ATR inhibitor combinatorial therapy with other DNA damaging agents or PARP inhibitors have shown great promise with high degrees of cytotoxicity in cancer cells. However, some mixed results were also reported, where patient and mouse pharmacokinetic data suggest that ATR inhibitors exhibit a sharp decrease in plasma concentration [150], leading to only a short burst of full ATR inhibition [151]. Additionally, adverse effects have also been reported, potentially due to the multiple roles that ATR has throughout the cell cycle. For instance, the very first ATR inhibitor to enter clinical trials, BAY 1895344, was discontinued due to both disease progression in patients and adverse side effects [152]. In another clinical trial, patients given the ATR inhibitor gartisertib showed increased blood bilirubin when used as a single agent or when combined with carboplatin [153]. Combination therapy of berzosertib, gemcitabine, and cisplatin led to high neutropenia and thrombocytopenia in a different clinical trial as well [154]. Finally, another ATR inhibitor in clinical trials, Ceralasertib, has also shown instances of thrombocytopenia, neutropenia, and anaemia in some patients. Therefore, while ATR inhibitors show great promise in cancer therapeutics, it is critical to assess why these adverse effects occur in the light of emerging non-canonical roles of ATR. More in-depth reviews on the efficacies and progress of ATR inhibitors in clinical trials are reviewed extensively in other recent reviews [17,154].

## 5. Conclusions

Separate from its role in the detection of replication stress and ssDNA breaks, ATR has a role in mitosis, where it is recruited to centromeric R-loops. Once recruited, ATR is potentially activated by ETAA1, which then promotes Aurora B activity and chromosome segregation even in the absence of DNA damage. ATR also has a close association with the nuclear membrane, where it not only detects membrane deformations and mechanical stress but can also affect phosphorylation, both directly and indirectly, of Lamin A to regulate membrane integrity and rupture in primary nuclei (PN) and micronuclei (MN). Finally, ATR also has an unexpected role in promoting DAXX association with PML bodies to regulate CENP-A and histone H3.3 occupancy at the centromere, which is another mechanism by which ATR promotes faithful chromosome segregation during mitosis. The relationship between ATR and CENP-A may be even more complex, as CENP-A affects ATR activation at centromeres during replication [155,156]. It is likely that even more novel roles of ATR will be discovered, and this may shed more light on the multifaceted functions of this kinase. Indeed, it is possible that because of these multiple critical roles, ATR knockout is inviable unlike other PI3 kinases including ATM [157,158]. Likewise, ATR is not as commonly mutated or depleted in cancer cells as has been observed for ATM (TCGA).

With these newly identified functions, it is important to reevaluate the clinical strategies and implications of ATR inhibitors as a cancer therapy. As mentioned earlier in this review, the premise and promise of ATR as a target of cancer therapy is based on the idea of synthetic lethality. Since a large subset of solid tumors have DNA damage repair deficiency and chromosomal instability (CIN) phenotypes, further impairing the DDR machinery through ATR inhibition would lead to intolerable amounts of DNA damage within these cancer cells, selectively killing cancer cells over healthy cells. While this is a sound theory, it is extremely difficult to evaluate whether ATR inhibition, which will also disrupt the non-canonical pathways discussed above, would be beneficial or harmful. For instance, ATR inhibition would cause reduced micronuclei rupture, which could prevent chromothripsis and chromosomal rearrangements, but at the same time promote proliferation of cells that harbor abnormal numbers of chromosomes. Likewise, it is difficult to assess whether artificially changing the distribution of CENP-A and histone H3.3 at the centromeres by inhibition of ATR is beneficial or harmful to cancerous and healthy cells. Perhaps the high toxicity and side effects of ATR inhibitors that occur in patients in clinical trials are due to the lack of knowledge of the consequences of affecting these non-canonical pathways. Thus, more studies should be conducted to fully understand the physiological consequences of ATR inhibition and how best to target key aspects of ATR activity. 

## References

[1] Bahrami H. (2024). Interpreting Cancer Incidence Rates and Trends: A Review of Control Factors and Worldwide Statistics. J. Cancer Res. Pract..

[2] Hosea R., Hillary S., Naqvi S., Wu S., Kasim V. (2024). The two sides of chromosomal instability: Drivers and brakes in cancer. Signal Transduct. Target. Ther..

[3] Thompson S.L., Bakhoum S.F., Compton D.A. (2010). Mechanisms of chromosomal instability. Curr. Biol..

[4] Bakhoum S.F., Cantley L.C. (2018). The Multifaceted Role of Chromosomal Instability in Cancer and Its Microenvironment. Cell.

[5] Zhang C.Z., Spektor A., Cornils H., Francis J.M., Jackson E.K., Liu S., Meyerson M., Pellman D. (2015). Chromothripsis from DNA damage in micronuclei. Nature.

[6] Hatch E.M., Hetzer M.W. (2015). Linking Micronuclei to Chromosome Fragmentation. Cell.

[7] Burrell R.A., McClelland S.E., Endesfelder D., Groth P., Weller M.C., Shaikh N., Domingo E., Kanu N., Dewhurst S.M., Gronroos E. (2013). Replication stress links structural and numerical cancer chromosomal instability. Nature.

[8] Chan K.L., North P.S., Hickson I.D. (2007). BLM is required for faithful chromosome segregation and its localization defines a class of ultrafine anaphase bridges. EMBO J..

[9] Yin M., Hong F., Wang Q.E., Sergi C.M. (2022). DNA Damage Response and Cancer Metastasis: Clinical Implications and Therapeutic Opportunities. Metastasis.

[10] Reinhardt H.C., Aslanian A.S., Lees J.A., Yaffe M.B. (2007). p53-deficient cells rely on ATM- and ATR-mediated checkpoint signaling through the p38MAPK/MK2 pathway for survival after DNA damage. Cancer Cell.

[11] Lecona E., Fernandez-Capetillo O. (2018). Targeting ATR in cancer. Nat. Rev. Cancer.

[12] Hopkins J.L., Lan L., Zou L. (2022). DNA repair defects in cancer and therapeutic opportunities. Genes. Dev..

[13] Lavin M.F. (2008). Ataxia-telangiectasia: From a rare disorder to a paradigm for cell signalling and cancer. Nat. Rev. Mol. Cell Biol..

[14] Tanaka A., Weinel S., Nagy N., O’Driscoll M., Lai-Cheong J.E., Kulp-Shorten C.L., Knable A., Carpenter G., Fisher S.A., Hiragun M. (2012). Germline mutation in ATR in autosomal- dominant oropharyngeal cancer syndrome. Am. J. Hum. Genet..

[15] Wengner A.M., Siemeister G., Lucking U., Lefranc J., Wortmann L., Lienau P., Bader B., Bomer U., Moosmayer D., Eberspacher U. (2020). The Novel ATR Inhibitor BAY 1895344 Is Efficacious as Monotherapy and Combined with DNA Damage-Inducing or Repair-Compromising Therapies in Preclinical Cancer Models. Mol. Cancer Ther..

[16] O’Brien S., Ubhi T., Wolf L., Gandhi K., Lin S., Chaudary N., Dhani N.C., Milosevic M., Brown G.W., Angers S. (2023). FBXW7-loss Sensitizes Cells to ATR Inhibition Through Induced Mitotic Catastrophe. Cancer Res. Commun..

[17] Yano K., Shiotani B. (2023). Emerging strategies for cancer therapy by ATR inhibitors. Cancer Sci..

[18] Murga M., Campaner S., Lopez-Contreras A.J., Toledo L.I., Soria R., Montana M.F., Artista L., Schleker T., Guerra C., Garcia E. (2011). Exploiting oncogene-induced replicative stress for the selective killing of Myc-driven tumors. Nat. Struct. Mol. Biol..

[19] Middleton M.R., Dean E., Evans T.R.J., Shapiro G.I., Pollard J., Hendriks B.S., Falk M., Diaz-Padilla I., Plummer R. (2021). Phase 1 study of the ATR inhibitor berzosertib (formerly M6620, VX-970) combined with gemcitabine +/- cisplatin in patients with advanced solid tumours. Br. J. Cancer.

[20] Yap T.A., Krebs M.G., Postel-Vinay S., El-Khouiery A., Soria J.C., Lopez J., Berges A., Cheung S.Y.A., Irurzun-Arana I., Goldwin A. (2021). Ceralasertib (AZD6738), an Oral ATR Kinase Inhibitor, in Combination with Carboplatin in Patients with Advanced Solid Tumors: A Phase I Study. Clin. Cancer Res..

[21] Martorana F., Da Silva L.A., Sessa C., Colombo I. (2022). Everything Comes with a Price: The Toxicity Profile of DNA-Damage Response Targeting Agents. Cancers.

[22] O’Driscoll M. (2012). Diseases associated with defective responses to DNA damage. Cold Spring Harb. Perspect. Biol..

[23] O’Driscoll M., Jackson A.P., Jeggo P.A. (2006). Microcephalin: A causal link between impaired damage response signalling and microcephaly. Cell Cycle.

[24] Barlow C., Dennery P.A., Shigenaga M.K., Smith M.A., Morrow J.D., Roberts L.J., Wynshaw-Boris A., Levine R.L. (1999). Loss of the ataxia-telangiectasia gene product causes oxidative damage in target organs. Proc. Natl. Acad. Sci. USA.

[25] McConnell M.J., Kaushal D., Yang A.H., Kingsbury M.A., Rehen S.K., Treuner K., Helton R., Annas E.G., Chun J., Barlow C. (2004). Failed clearance of aneuploid embryonic neural progenitor cells leads to excess aneuploidy in the Atm-deficient but not the Trp53-deficient adult cerebral cortex. J. Neurosci..

[26] Rass U., Ahel I., West S.C. (2007). Defective DNA repair and neurodegenerative disease. Cell.

[27] Reynolds J.J., Stewart G.S. (2013). A single strand that links multiple neuropathologies in human disease. Brain.

[28] Fred C.L. (2022). The DNA damage response—From cell biology to human disease. J. Transl. Genet. Genom..

[29] Kirtay M., Sell J., Marx C., Haselmann H., Ceanga M., Zhou Z.W., Rahmati V., Kirkpatrick J., Buder K., Grigaravicius P. (2021). ATR regulates neuronal activity by modulating presynaptic firing. Nat. Commun..

[30] Saldivar J.C., Cortez D., Cimprich K.A. (2017). The essential kinase ATR: Ensuring faithful duplication of a challenging genome. Nat. Rev. Mol. Cell Biol..

[31] Cimprich K.A., Cortez D. (2008). ATR: An essential regulator of genome integrity. Nat. Rev. Mol. Cell Biol..

[32] Nam E.A., Cortez D. (2011). ATR signalling: More than meeting at the fork. Biochem. J..

[33] Lovejoy C.A., Cortez D. (2009). Common mechanisms of PIKK regulation. DNA Repair..

[34] Mordes D.A., Cortez D. (2008). Activation of ATR and related PIKKs. Cell Cycle.

[35] Falck J., Coates J., Jackson S.P. (2005). Conserved modes of recruitment of ATM, ATR and DNA-PKcs to sites of DNA damage. Nature.

[36] Zou L., Elledge S.J. (2003). Sensing DNA damage through ATRIP recognition of RPA-ssDNA complexes. Science.

[37] Singleton B.K., Torres-Arzayus M.I., Rottinghaus S.T., Taccioli G.E., Jeggo P.A. (1999). The C terminus of Ku80 activates the DNA-dependent protein kinase catalytic subunit. Mol. Cell. Biol..

[38] Blackford A.N., Jackson S.P. (2017). ATM, ATR, and DNA-PK: The Trinity at the Heart of the DNA Damage Response. Mol. Cell.

[39] Edwards R.J., Bentley N.J., Carr A.M. (1999). A Rad3–Rad26 complex responds to DNA damage independently of other checkpoint proteins. Nat. Cell Biol..

[40] Ball H.L., Myers J.S., Cortez D. (2005). ATRIP binding to replication protein A-single-stranded DNA promotes ATR-ATRIP localization but is dispensable for Chk1 phosphorylation. Mol. Biol. Cell.

[41] Symington L.S. (2014). End resection at double-strand breaks: Mechanism and regulation. Cold Spring Harb. Perspect. Biol..

[42] Bantele S.C.S., Lisby M., Pfander B. (2019). Quantitative sensing and signalling of single-stranded DNA during the DNA damage response. Nat. Commun..

[43] Marechal A., Zou L. (2013). DNA damage sensing by the ATM and ATR kinases. Cold Spring Harb. Perspect. Biol..

[44] Lin Y., Li J., Zhao H., McMahon A., McGhee K., Yan S. (2023). APE1 recruits ATRIP to ssDNA in an RPA-dependent and -independent manner to promote the ATR DNA damage response. Elife.

[45] Burrows A.E., Elledge S.J. (2008). How ATR turns on: TopBP1 goes on ATRIP with ATR. Genes. Dev..

[46] Yan S., Michael W.M. (2009). TopBP1 and DNA polymerase-α directly recruit the 9-1-1 complex to stalled DNA replication forks. J. Cell Biol..

[47] Parrilla-Castellar E.R., Arlander S.J.H., Karnitz L. (2004). Dial 9–1–1 for DNA damage: The Rad9–Hus1–Rad1 (9–1–1) clamp complex. DNA Repair..

[48] Kim H.S., Brill S.J. (2001). Rfc4 interacts with Rpa1 and is required for both DNA replication and DNA damage checkpoints in Saccharomyces cerevisiae. Mol. Cell. Biol..

[49] Rappas M., Oliver A.W., Pearl L.H. (2011). Structure and function of the Rad9-binding region of the DNA-damage checkpoint adaptor TopBP1. Nucleic Acids Res..

[50] Navadgi-Patil V.M., Burgers P.M. (2009). A tale of two tails: Activation of DNA damage checkpoint kinase Mec1/ATR by the 9-1-1 clamp and by Dpb11/TopBP1. DNA Repair..

[51] Delacroix S., Wagner J.M., Kobayashi M., Yamamoto K., Karnitz L.M. (2007). The Rad9-Hus1-Rad1 (9-1-1) clamp activates checkpoint signaling via TopBP1. Genes. Dev..

[52] Lee J., Dunphy W.G. (2010). Rad17 plays a central role in establishment of the interaction between TopBP1 and the Rad9-Hus1-Rad1 complex at stalled replication forks. Mol. Biol. Cell.

[53] Kumagai A., Lee J., Yoo H.Y., Dunphy W.G. (2006). TopBP1 activates the ATR-ATRIP complex. Cell.

[54] Duursma A.M., Driscoll R., Elias J.E., Cimprich K.A. (2013). A role for the MRN complex in ATR activation via TOPBP1 recruitment. Mol. Cell.

[55] Ramirez-Lugo J.S., Yoo H.Y., Yoon S.J., Dunphy W.G. (2011). CtIP interacts with TopBP1 and Nbs1 in the response to double-stranded DNA breaks (DSBs) in Xenopus egg extracts. Cell Cycle.

[56] Gong Z., Kim J.E., Leung C.C., Glover J.N., Chen J. (2010). BACH1/FANCJ acts with TopBP1 and participates early in DNA replication checkpoint control. Mol. Cell.

[57] Haahr P., Hoffmann S., Tollenaere M.A., Ho T., Toledo L.I., Mann M., Bekker-Jensen S., Raschle M., Mailand N. (2016). Activation of the ATR kinase by the RPA-binding protein ETAA1. Nat. Cell Biol..

[58] Bass T.E., Luzwick J.W., Kavanaugh G., Carroll C., Dungrawala H., Glick G.G., Feldkamp M.D., Putney R., Chazin W.J., Cortez D. (2016). ETAA1 acts at stalled replication forks to maintain genome integrity. Nat. Cell Biol..

[59] Lee Y.C., Zhou Q., Chen J., Yuan J. (2016). RPA-Binding Protein ETAA1 Is an ATR Activator Involved in DNA Replication Stress Response. Curr. Biol..

[60] Achuthankutty D., Thakur R.S., Haahr P., Hoffmann S., Drainas A.P., Bizard A.H., Weischenfeldt J., Hickson I.D., Mailand N. (2019). Regulation of ETAA1-mediated ATR activation couples DNA replication fidelity and genome stability. J. Cell Biol..

[61] Zhao H., Piwnica-Worms H. (2001). ATR-mediated checkpoint pathways regulate phosphorylation and activation of human Chk1. Mol. Cell. Biol..

[62] Kumagai A., Dunphy W.G. (2000). Claspin, a novel protein required for the activation of Chk1 during a DNA replication checkpoint response in Xenopus egg extracts. Mol. Cell.

[63] Liu S., Bekker-Jensen S., Mailand N., Lukas C., Bartek J., Lukas J. (2006). Claspin operates downstream of TopBP1 to direct ATR signaling towards Chk1 activation. Mol. Cell. Biol..

[64] Gorecki L., Andrs M., Korabecny J. (2021). Clinical Candidates Targeting the ATR-CHK1-WEE1 Axis in Cancer. Cancers.

[65] Liu Q., Guntuku S., Cui X.S., Matsuoka S., Cortez D., Tamai K., Luo G., Carattini-Rivera S., DeMayo F., Bradley A. (2000). Chk1 is an essential kinase that is regulated by Atr and required for the G(2)/M DNA damage checkpoint. Genes. Dev..

[66] Kumagai A., Guo Z., Emami K.H., Wang S.X., Dunphy W.G. (1998). The Xenopus Chk1 Protein Kinase Mediates a Caffeine-sensitive Pathway of Checkpoint Control in Cell-free Extracts. J. Cell Biol..

[67] Royou A., McCusker D., Kellogg D.R., Sullivan W. (2008). Grapes(Chk1) prevents nuclear CDK1 activation by delaying cyclin B nuclear accumulation. J. Cell Biol..

[68] Gould K.L., Nurse P. (1989). Tyrosine phosphorylation of the fission yeast cdc2+ protein kinase regulates entry into mitosis. Nature.

[69] Wang Q., Bode A.M., Zhang T. (2023). Targeting CDK1 in cancer: Mechanisms and implications. NPJ Precis. Oncol..

[70] Vassilev L.T. (2006). Cell cycle synchronization at the G2/M phase border by reversible inhibition of CDK1. Cell Cycle.

[71] Purdy A., Uyetake L., Cordeiro M.G., Su T.T. (2005). Regulation of mitosis in response to damaged or incompletely replicated DNA require different levels of Grapes (Drosophila Chk1). J. Cell Sci..

[72] Ou Y.H., Chung P.H., Sun T.P., Shieh S.Y. (2005). p53 C-terminal phosphorylation by CHK1 and CHK2 participates in the regulation of DNA-damage-induced C-terminal acetylation. Mol. Biol. Cell.

[73] Saldivar J.C., Hamperl S., Bocek M.J., Chung M., Bass T.E., Cisneros-Soberanis F., Samejima K., Xie L., Paulson J.R., Earnshaw W.C. (2018). An intrinsic S/G2 checkpoint enforced by ATR. Science.

[74] Peng C.Y., Graves P.R., Thoma R.S., Wu Z., Shaw A.S., Piwnica-Worms H. (1997). Mitotic and G2 checkpoint control: Regulation of 14-3-3 protein binding by phosphorylation of Cdc25C on serine-216. Science.

[75] Dimitrova D.S., Gilbert D.M. (2000). Temporally coordinated assembly and disassembly of replication factories in the absence of DNA synthesis. Nat. Cell Biol..

[76] Moiseeva T., Hood B., Schamus S., O’Connor M.J., Conrads T.P., Bakkenist C.J. (2017). ATR kinase inhibition induces unscheduled origin firing through a Cdc7-dependent association between GINS and And-1. Nat. Commun..

[77] Mirkin E.V., Mirkin S.M. (2007). Replication fork stalling at natural impediments. Microbiol. Mol. Biol. Rev..

[78] Ge X.Q., Blow J.J. (2010). Chk1 inhibits replication factory activation but allows dormant origin firing in existing factories. J. Cell Biol..

[79] Blow J.J., Ge X.Q. (2009). A model for DNA replication showing how dormant origins safeguard against replication fork failure. EMBO Rep..

[80] Moiseeva T.N., Yin Y., Calderon M.J., Qian C., Schamus-Haynes S., Sugitani N., Osmanbeyoglu H.U., Rothenberg E., Watkins S.C., Bakkenist C.J. (2019). An ATR and CHK1 kinase signaling mechanism that limits origin firing during unperturbed DNA replication. Proc. Natl. Acad. Sci. USA.

[81] Trenz K., Smith E., Smith S., Costanzo V. (2006). ATM and ATR promote Mre11 dependent restart of collapsed replication forks and prevent accumulation of DNA breaks. EMBO J..

[82] Wang H., Guan J., Wang H., Perrault A.R., Wang Y., Iliakis G. (2001). Replication protein A2 phosphorylation after DNA damage by the coordinated action of ataxia telangiectasia-mutated and DNA-dependent protein kinase. Cancer Res..

[83] Rankin B.D., Rankin S. (2024). The MCM2-7 Complex: Roles beyond DNA Unwinding. Biology.

[84] Liu H., Takeda S., Kumar R., Westergard T.D., Brown E.J., Pandita T.K., Cheng E.H., Hsieh J.J. (2010). Phosphorylation of MLL by ATR is required for execution of mammalian S-phase checkpoint. Nature.

[85] Liu H., Takeda S., Cheng E.H., Hsieh J.J. (2008). Biphasic MLL takes helm at cell cycle control: Implications in human mixed lineage leukemia. Cell Cycle.

[86] Trenz K., Errico A., Costanzo V. (2008). Plx1 is required for chromosomal DNA replication under stressful conditions. EMBO J..

[87] Yoo H.Y., Shevchenko A., Shevchenko A., Dunphy W.G. (2004). Mcm2 is a direct substrate of ATM and ATR during DNA damage and DNA replication checkpoint responses. J. Biol. Chem..

[88] Byun T.S., Pacek M., Yee M.C., Walter J.C., Cimprich K.A. (2005). Functional uncoupling of MCM helicase and DNA polymerase activities activates the ATR-dependent checkpoint. Genes. Dev..

[89] Ibarra A., Schwob E., Mendez J. (2008). Excess MCM proteins protect human cells from replicative stress by licensing backup origins of replication. Proc. Natl. Acad. Sci. USA.

[90] Woodward A.M., Gohler T., Luciani M.G., Oehlmann M., Ge X., Gartner A., Jackson D.A., Blow J.J. (2006). Excess Mcm2-7 license dormant origins of replication that can be used under conditions of replicative stress. J. Cell Biol..

[91] Tsvetkov L., Stern D.F. (2005). Interaction of chromatin-associated Plk1 and Mcm7. J. Biol. Chem..

[92] van Vugt M.A., Gardino A.K., Linding R., Ostheimer G.J., Reinhardt H.C., Ong S.E., Tan C.S., Miao H., Keezer S.M., Li J. (2010). A mitotic phosphorylation feedback network connects Cdk1, Plk1, 53BP1, and Chk2 to inactivate the G(2)/M DNA damage checkpoint. PLoS Biol..

[93] Song B., Liu X.S., Liu X. (2012). Polo-like kinase 1 (Plk1): An Unexpected Player in DNA Replication. Cell Div..

[94] Courtot L., Hoffmann J.S., Bergoglio V. (2018). The Protective Role of Dormant Origins in Response to Replicative Stress. Int. J. Mol. Sci..

[95] Giunta S., Belotserkovskaya R., Jackson S.P. (2010). DNA damage signaling in response to double-strand breaks during mitosis. J. Cell Biol..

[96] Nelson G., Buhmann M., von Zglinicki T. (2009). DNA damage foci in mitosis are devoid of 53BP1. Cell Cycle.

[97] Peterson S.E., Li Y., Chait B.T., Gottesman M.E., Baer R., Gautier J. (2011). Cdk1 uncouples CtIP-dependent resection and Rad51 filament formation during M-phase double-strand break repair. J. Cell Biol..

[98] Lee D.H., Acharya S.S., Kwon M., Drane P., Guan Y., Adelmant G., Kalev P., Shah J., Pellman D., Marto J.A. (2014). Dephosphorylation enables the recruitment of 53BP1 to double-strand DNA breaks. Mol. Cell.

[99] Blackford A.N., Stucki M. (2020). How Cells Respond to DNA Breaks in Mitosis. Trends Biochem. Sci..

[100] Shiotani B., Zou L. (2009). Single-stranded DNA orchestrates an ATM-to-ATR switch at DNA breaks. Mol. Cell.

[101] Ammazzalorso F., Pirzio L.M., Bignami M., Franchitto A., Pichierri P. (2010). ATR and ATM differently regulate WRN to prevent DSBs at stalled replication forks and promote replication fork recovery. EMBO J..

[102] Kabeche L., Nguyen H.D., Buisson R., Zou L. (2018). A mitosis-specific and R loop-driven ATR pathway promotes faithful chromosome segregation. Science.

[103] Belotserkovskii B.P., Tornaletti S., D’Souza A.D., Hanawalt P.C. (2018). R-loop generation during transcription: Formation, processing and cellular outcomes. DNA Repair..

[104] Ariel F., Lucero L., Christ A., Mammarella M.F., Jegu T., Veluchamy A., Mariappan K., Latrasse D., Blein T., Liu C. (2020). R-Loop Mediated trans Action of the APOLO Long Noncoding RNA. Mol. Cell.

[105] Toriumi K., Tsukahara T., Hanai R. (2013). R-Loop Formation In Trans at an AGGAG Repeat. J. Nucleic Acids.

[106] Palozola K.C., Liu H., Nicetto D., Zaret K.S. (2017). Low-Level, Global Transcription during Mitosis and Dynamic Gene Reactivation during Mitotic Exit. Cold Spring Harb. Symp. Quant. Biol..

[107] Chan F.L., Marshall O.J., Saffery R., Kim B.W., Earle E., Choo K.H., Wong L.H. (2012). Active transcription and essential role of RNA polymerase II at the centromere during mitosis. Proc. Natl. Acad. Sci. USA.

[108] Barra V., Fachinetti D. (2018). The dark side of centromeres: Types, causes and consequences of structural abnormalities implicating centromeric DNA. Nat. Commun..

[109] Guerrero A.A., Gamero M.C., Trachana V., Futterer A., Pacios-Bras C., Diaz-Concha N.P., Cigudosa J.C., Martinez A.C., van Wely K.H. (2010). Centromere-localized breaks indicate the generation of DNA damage by the mitotic spindle. Proc. Natl. Acad. Sci. USA.

[110] Min J., Wright W.E., Shay J.W. (2017). Alternative Lengthening of Telomeres Mediated by Mitotic DNA Synthesis Engages Break-Induced Replication Processes. Mol. Cell. Biol..

[111] Bang S.W., Ko M.J., Kang S., Kim G.S., Kang D., Lee J., Hwang D.S. (2011). Human TopBP1 localization to the mitotic centrosome mediates mitotic progression. Exp. Cell Res..

[112] Bagge J., Oestergaard V.H., Lisby M. (2021). Functions of TopBP1 in preserving genome integrity during mitosis. Semin. Cell Dev. Biol..

[113] Ummethum H., Li J., Lisby M., Oestergaard V.H. (2023). Emerging roles of the CIP2A-TopBP1 complex in genome integrity. NAR Cancer.

[114] Perera D., Perez-Hidalgo L., Moens P.B., Reini K., Lakin N., Syvaoja J.E., San-Segundo P.A., Freire R. (2004). TopBP1 and ATR colocalization at meiotic chromosomes: Role of TopBP1/Cut5 in the meiotic recombination checkpoint. Mol. Biol. Cell.

[115] Reini K., Uitto L., Perera D., Moens P.B., Freire R., Syvaoja J.E. (2004). TopBP1 localises to centrosomes in mitosis and to chromosome cores in meiosis. Chromosoma.

[116] Lyu K., Kumagai A., Dunphy W.G. (2019). RPA-coated single-stranded DNA promotes the ETAA1-dependent activation of ATR. Cell Cycle.

[117] Bass T.E., Cortez D. (2019). Quantitative phosphoproteomics reveals mitotic function of the ATR activator ETAA1. J. Cell Biol..

[118] Gonzalez Besteiro M.A., Gottifredi V. (2019). ETAA1 ensures proper chromosome segregation: A matter of S phase or mitosis?. J. Cell Biol..

[119] Thada V., Cortez D. (2019). Common motifs in ETAA1 and TOPBP1 required for ATR kinase activation. J. Biol. Chem..

[120] Jiang M., Zhao L., Gamez M., Imperiale M.J. (2012). Roles of ATM and ATR-mediated DNA damage responses during lytic BK polyomavirus infection. PLoS Pathog..

[121] Brown E.J., Baltimore D. (2003). Essential and dispensable roles of ATR in cell cycle arrest and genome maintenance. Genes. Dev..

[122] Chen X., Zhao R., Glick G.G., Cortez D. (2007). Function of the ATR N-terminal domain revealed by an ATM/ATR chimera. Exp. Cell Res..

[123] Williams R.M., Yates L.A., Zhang X. (2020). Structures and regulations of ATM and ATR, master kinases in genome integrity. Curr. Opin. Struct. Biol..

[124] Zachos G., Black E.J., Walker M., Scott M.T., Vagnarelli P., Earnshaw W.C., Gillespie D.A. (2007). Chk1 is required for spindle checkpoint function. Dev. Cell.

[125] Hatch E.M., Hetzer M.W. (2016). Nuclear envelope rupture is induced by actin-based nucleus confinement. J. Cell Biol..

[126] Zhang Q., Tamashunas A.C., Agrawal A., Torbati M., Katiyar A., Dickinson R.B., Lammerding J., Lele T.P. (2019). Local, transient tensile stress on the nuclear membrane causes membrane rupture. Mol. Biol. Cell.

[127] Kidiyoor G.R., Li Q., Bastianello G., Bruhn C., Giovannetti I., Mohamood A., Beznoussenko G.V., Mironov A., Raab M., Piel M. (2020). ATR is essential for preservation of cell mechanics and nuclear integrity during interstitial migration. Nat. Commun..

[128] Kumar A., Mazzanti M., Mistrik M., Kosar M., Beznoussenko G.V., Mironov A.A., Garre M., Parazzoli D., Shivashankar G.V., Scita G. (2014). ATR mediates a checkpoint at the nuclear envelope in response to mechanical stress. Cell.

[129] Xia Y., Pfeifer C.R., Cho S., Discher D.E., Irianto J. (2018). Nuclear mechanosensing. Emerg. Top. Life Sci..

[130] Kovacs M.T., Vallette M., Wiertsema P., Dingli F., Loew D., Nader G.P.F., Piel M., Ceccaldi R. (2023). DNA damage induces nuclear envelope rupture through ATR-mediated phosphorylation of lamin A/C. Mol. Cell.

[131] Joo Y.K., Black E.M., Trier I., Haakma W., Zou L., Kabeche L. (2023). ATR promotes clearance of damaged DNA and damaged cells by rupturing micronuclei. Mol. Cell.

[132] Krupina K., Goginashvili A., Cleveland D.W. (2021). Causes and consequences of micronuclei. Curr. Opin. Cell Biol..

[133] Liu S., Shiotani B., Lahiri M., Marechal A., Tse A., Leung C.C., Glover J.N., Yang X.H., Zou L. (2011). ATR autophosphorylation as a molecular switch for checkpoint activation. Mol. Cell.

[134] Adhikari D., Zheng W., Shen Y., Gorre N., Ning Y., Halet G., Kaldis P., Liu K. (2012). Cdk1, but not Cdk2, is the sole Cdk that is essential and sufficient to drive resumption of meiosis in mouse oocytes. Hum. Mol. Genet..

[135] Hamirally S., Kamil J.P., Ndassa-Colday Y.M., Lin A.J., Jahng W.J., Baek M.C., Noton S., Silva L.A., Simpson-Holley M., Knipe D.M. (2009). Viral mimicry of Cdc2/cyclin-dependent kinase 1 mediates disruption of nuclear lamina during human cytomegalovirus nuclear egress. PLoS Pathog..

[136] Reimann H., Stopper H., Hintzsche H. (2023). Fate of micronuclei and micronucleated cells after treatment of HeLa cells with different genotoxic agents. Arch. Toxicol..

[137] Kneissig M., Keuper K., de Pagter M.S., van Roosmalen M.J., Martin J., Otto H., Passerini V., Campos Sparr A., Renkens I., Kropveld F. (2019). Micronuclei-based model system reveals functional consequences of chromothripsis in human cells. Elife.

[138] Maass K.K., Rosing F., Ronchi P., Willmund K.V., Devens F., Hergt M., Herrmann H., Lichter P., Ernst A. (2018). Altered nuclear envelope structure and proteasome function of micronuclei. Exp. Cell Res..

[139] Bischof O., Kim S.H., Irving J., Beresten S., Ellis N.A., Campisi J. (2001). Regulation and localization of the Bloom syndrome protein in response to DNA damage. J. Cell Biol..

[140] Carbone R., Pearson M., Minucci S., Pelicci P.G. (2002). PML NBs associate with the hMre11 complex and p53 at sites of irradiation induced DNA damage. Oncogene.

[141] Dellaire G., Bazett-Jones D.P. (2004). PML nuclear bodies: Dynamic sensors of DNA damage and cellular stress. Bioessays.

[142] Sahin U., Ferhi O., Jeanne M., Benhenda S., Berthier C., Jollivet F., Niwa-Kawakita M., Faklaris O., Setterblad N., de The H. (2014). Oxidative stress-induced assembly of PML nuclear bodies controls sumoylation of partner proteins. J. Cell Biol..

[143] Yau T.Y., Sander W., Eidson C., Courey A.J. (2021). SUMO Interacting Motifs: Structure and Function. Cells.

[144] Wu C.S., Ouyang J., Mori E., Nguyen H.D., Marechal A., Hallet A., Chen D.J., Zou L. (2014). SUMOylation of ATRIP potentiates DNA damage signaling by boosting multiple protein interactions in the ATR pathway. Genes. Dev..

[145] Barr S.M., Leung C.G., Chang E.E., Cimprich K.A. (2003). ATR kinase activity regulates the intranuclear translocation of ATR and RPA following ionizing radiation. Curr. Biol..

[146] Trier I., Black E.M., Joo Y.K., Kabeche L. (2023). ATR protects centromere identity by promoting DAXX association with PML nuclear bodies. Cell Rep..

[147] Gresko E., Ritterhoff S., Sevilla-Perez J., Roscic A., Frobius K., Kotevic I., Vichalkovski A., Hess D., Hemmings B.A., Schmitz M.L. (2009). PML tumor suppressor is regulated by HIPK2-mediated phosphorylation in response to DNA damage. Oncogene.

[148] Drane P., Ouararhni K., Depaux A., Shuaib M., Hamiche A. (2010). The death-associated protein DAXX is a novel histone chaperone involved in the replication-independent deposition of H3.3. Genes. Dev..

[149] Regnier V., Vagnarelli P., Fukagawa T., Zerjal T., Burns E., Trouche D., Earnshaw W., Brown W. (2005). CENP-A is required for accurate chromosome segregation and sustained kinetochore association of BubR1. Mol. Cell. Biol..

[150] Terranova N., Jansen M., Falk M., Hendriks B.S. (2021). Population pharmacokinetics of ATR inhibitor berzosertib in phase I studies for different cancer types. Cancer Chemother. Pharmacol..

[151] Chen T., Middleton F.K., Falcon S., Reaper P.M., Pollard J.R., Curtin N.J. (2015). Development of pharmacodynamic biomarkers for ATR inhibitors. Mol. Oncol..

[152] Yap T.A., Tan D.S.P., Terbuch A., Caldwell R., Guo C., Goh B.C., Heong V., Haris N.R.M., Bashir S., Drew Y. (2021). First-in-Human Trial of the Oral Ataxia Telangiectasia and RAD3-Related (ATR) Inhibitor BAY 1895344 in Patients with Advanced Solid Tumors. Cancer Discov..

[153] Burris H.A., Berlin J., Arkenau T., Cote G.M., Lolkema M.P., Ferrer-Playan J., Kalapur A., Bolleddula J., Locatelli G., Goddemeier T. (2024). A phase I study of ATR inhibitor gartisertib (M4344) as a single agent and in combination with carboplatin in patients with advanced solid tumours. Br. J. Cancer.

[154] Barnieh F.M., Loadman P.M., Falconer R.A. (2021). Progress towards a clinically-successful ATR inhibitor for cancer therapy. Curr. Res. Pharmacol. Drug Discov..

[155] Giunta S., Herve S., White R.R., Wilhelm T., Dumont M., Scelfo A., Gamba R., Wong C.K., Rancati G., Smogorzewska A. (2021). CENP-A chromatin prevents replication stress at centromeres to avoid structural aneuploidy. Proc. Natl. Acad. Sci. USA.

[156] Scelfo A., Angrisani A., Grillo M., Barnes B.M., Muyas F., Sauer C.M., Leung C.W.B., Dumont M., Grison M., Mazaud D. (2024). Specialized replication mechanisms maintain genome stability at human centromeres. Mol. Cell.

[157] Barlow C., Hirotsune S., Paylor R., Liyanage M., Eckhaus M., Collins F., Shiloh Y., Crawley J.N., Ried T., Tagle D. (1996). Atm-Deficient Mice: A Paradigm of Ataxia Telangiectasia. Cell.

[158] de Klein A., Muijtjens M., van Os R., Verhoeven Y., Smit B., Carr A.M., Lehmann A.R., Hoeijmakers J.H.J. (2000). Targeted disruption of the cell-cycle checkpoint gene ATR leads to early embryonic lethality in mice. Curr. Biol..

